# Animal Arterivirus Infections 

**DOI:** 10.1155/2014/303841

**Published:** 2014-06-23

**Authors:** Denis Archambault, Udeni B. R. Balasuriya, Raymond R. R. Rowland, Hanchun Yang, Dongwan Yoo

**Affiliations:** ^1^Department of Biological Sciences, University of Quebec at Montreal, P.O. Box 8888, Succursale Centre-Ville, Montreal, QC, Canada H3C 3P8; ^2^Department of Veterinary Science, University of Kentucky, 108 Maxwell H. Gluck Equine Research Center, Lexington, KY 40546-0099, USA; ^3^Department of Diagnostic Medicine and Pathobiology, College of Veterinary Medicine, Kansas State University Mosier Hall K-231, 1800 Denison Avenue, Manhattan, KS 66506, USA; ^4^Key Laboratory of Zoonosis of Ministry of Agriculture, College of Veterinary Medicine and State Key Laboratory of Agrobiotechnology, China Agricultural University, No. 2 Yuanmingyuan West Road, Beijing 100193, China; ^5^College of Veterinary Medicine, University of Illinois at Urbana-Champaign, 2001 South Lincoln Avenue, Veterinary Medicine Basic Sciences Building, Urbana, IL 61802, USA

The arteriviruses (Family* Arteriviridae*, Genus* Arterivirus*) include an interesting group of enveloped positive stranded RNA viruses that infect domestic and wild animals and they share a strikingly similar genome organization and replication strategy to that of coronaviruses, but differ considerably in their genetic complexity and virion architecture. Until recently, arteriviruses included equine arteritis virus (EAV), porcine reproductive and respiratory syndrome virus (PRRSV), lactate dehydrogenase-elevating virus (LDV) of mice, and simian hemorrhagic fever virus (SHFV). Three of these viruses were first discovered and characterized a long time ago (EAV-1953, LDV-1960 and SHFV-1964), whereas PRRSV emerged first in Europe and North America in the late 1980s and early 1990s. In 2012, arteriviruses were expanded to include the newly identified wobbly possum disease virus (WPDV) that causes neurologic disease among free-ranging Australian brushtail possums (*Trichosurus vulpecula*) in New Zealand. Similarly, four new genetically divergent SHFV variants were recently identified in a single male colobus monkey (*Procolobus rufomitratus tephrosceles*) and in African red-tailed (guenon) monkeys (*Cercopithecus ascanius*) from Kibale National Park, Uganda.

The arteriviruses are highly species specific, but share many biological and molecular properties, including virion morphology, a unique set of structural proteins, genome organization and replication strategy, and the ability to establish prolonged or true persistent infection in their natural hosts. However, the epidemiology and pathogenesis of the infections caused by each virus is distinct, as are the diseases they cause. EAV and PRRSV cause respiratory and reproductive disease in equids and swine, respectively. The natural hosts of LDV are field and the laboratory mice, but unlike other arteriviruses, LDV is generally non-pathogenic. Indeed this virus is a significant adventitious agent in the laboratory mice, and causes an increase of lactate dehydrogenase enzyme, contamination of transplantable tumors, clinically silent infection with mild pathology, and immunomodulation. SHFV establishes persistent, perhaps life long infections without disease in Patas monkeys (*Erythrocebus patas*), baboons (*Papio anuibus*), and African green monkeys (*Ceropithecus aethiops*). SHFVs of African origin are highly infectious and fatal in Asian rhesus monkeys (*Macacca mulatta, M. arctoides and M. fascicularis*). Although the human arterivirus is yet to be identified, there is significant concern about cross species transmission of some of these arteriviruses following xenotransplantation. There is no indication that any humans are infected with SHFV or any other arterivirus during disease outbreaks. However, it is obvious that transplantation of humans with tissues from SHFV-infected baboons or PRRSV-infected pigs would result in transfer of a considerable amount of these viruses, which could result in selection of a variant(s) that can replicate in humans. In this respect, PRRSV and SHFV should be seriously considered as a threat to xenotransplantation of tissues and organs from pigs and baboons.

Of the five arteriviruses, EAV and PRRSV are the most economically important viruses and pose a significant threat to equine and swine industries, respectively. Both viruses are distributed throughout the world, but PRRSV infection has become the most serious infectious disease of the pigs costing over $560 million US dollars per year to the US swine producers. Genetic manipulation of full-length cDNA clones of these two viruses has become an especially important and widely used tool to study the biology, pathogenesis, and virulence determinants of both of these viruses. Due to the economic impact, there has been significant research interest in molecular characterization of PRRSV and its interaction with the host defense system. In conjunction with advances in PRRSV molecular virology, reverse genetics and immunology, more and more attention is being directed to development of safe and efficacious vaccines against PRRSV. However, development of effective vaccines against PRRSV infection has been a significant challenge due to the presence of two genotypes of the virus with rapid emergence of antigenic variants and delayed suboptimal neutralizing antibody responses to infection. Current modified live and killed vaccines have had limited success, prompting investigation of alternative approaches to design new and improved vaccines.

In this special issue of Animal Arterivirus Infections by BioMed Research International dedicated to recent advances in arterivirus research, we have assembled seven manuscripts that describe original research data on PRRSV and EAV. The articles cover characterization of innate immune response to PRRSV and EAV, genetic diversity of PRRSV strains circulating in China, interaction between the structural and nonstructural proteins of PRRSV with the host cellular factors, vaccination-induced protection against homologous and heterologous challenge, and development of a recombinant DNA vaccine against PRRSV. These articles provide new insight into better understanding immune responses to PRRSV and EAV in the context of disease as well as vaccine-induced protection, which will help to develop better vaccines to control the growing worldwide burden of disease from these agents in the future. Clearly a comprehensive research approach combining molecular virology, immunology, viral pathogenesis, and host genetics is required to unravel the complexities of virus-host interactions of these viruses. We believe that we have assembled basic knowledge that encompasses these complexities, describes technologies that have contributed to this knowledge, and identifies at least some of the major problems faced in attempting to further understand the virus-host interactions that result in disease. We would like to thank all the authors and co-authors who contributed to this special issue of Animal Arterivirus Infections, as well as the numerous reviewers who reviewed each and every paper in a timely manner and provided their input to improve the quality and clarity of the final product. We would also like to acknowledge that there are many other colleagues and scientists who are active in the field of arteriviruses whose expertise has not been represented here.

*Denis Archambault* *Udeni B. R. Balasuriya* *Raymond R. R. Rowland* *Hanchun Yang* *Dongwan Yoo*

